# Community Group Membership and Stigmatising Attitudes Towards People Living with HIV in Eastern Zimbabwe

**DOI:** 10.1007/s10900-013-9741-6

**Published:** 2013-08-03

**Authors:** Mercy Nhamo-Murire, Catherine Campbell, Simon Gregson

**Affiliations:** 1 Women’s University in Africa, Harare, Zimbabwe; 2 London School of Economics and Political Science, London, UK; 3 Imperial College London, London, UK; 4 Biomedical Research and Training Institute, Harare, Zimbabwe

**Keywords:** HIV and AIDS, Stigmatising attitudes, Community groups, Zimbabwe

## Abstract

Stigmatising attitudes towards people living with HIV and AIDS (PLHIV) are hampering attempts to control HIV epidemics in sub-Saharan African countries. This study measures the effect of social capital, in the form of local community groups, in reducing stigma and tests a new explanatory framework for the association between community group membership and less stigmatising attitudes. Prospective data on membership of a wide range of different community groups and stigmatising attitudes (being unwilling to care for a relative with AIDS), collected from a general population cohort of 5,253 men and women aged 15–54 years in eastern Zimbabwe between 2003 and 2008 were analysed using multivariable logistic regression. 36 % of respondents were members of community groups throughout the study period. Individuals in community groups were less likely to express stigmatising attitudes towards PLHIV—3.4 versus 9.5 % (adjusted odds ratio = 0.46, *p* < 0.001). Discussions of care for PLHIV within groups, improved knowledge about AIDS, greater exposure to PLHIV, and increased uptake of HIV testing and counselling did not account for the association. Further work is needed to identify the mechanisms through which community participation can reduce stigma. Nevertheless, these findings suggest that promoting well-informed discussions about HIV within pre-existing community groups and involving these groups in stigma reduction programmes could be effective means of reducing stigma at the grassroots level.

## Introduction

Stigmatising attitudes towards people living with HIV and AIDS (PLHIV) have long been one of the major problems facing infected individuals and hampering attempts to control HIV epidemics in Zimbabwe [16] and elsewhere [2]. A number of approaches to reducing stigmatising attitudes have been identified [29] but relatively few of these have been shown to be effective [14]. One promising but little explored approach is the possibility that social capital, in the form of community group participation, might be a useful resource in challenging and reducing stigma. Community groups are rooted in the local social contexts within which individuals form attitudes towards PLHIV and could help to reduce stigma by providing opportunities for discussion and renegotiation of previously stigmatising social norms. Community group memberships affect the formation of self-identity as well as a person’s attitudes to others [4]. Social capital has been found to be associated with reduced rates of HIV acquisition [8, 21, 36] and with a number of factors linked to the risk of infection including alcohol consumption [8], intimate partner violence [36] and sexual behaviour [11].

Community groups can provide the social support and psychological resources to ‘reconstrue’ threats to one’s sense of identity and well-being, such as the threat posed by the presence of large numbers of HIV-infected individuals in one’s social circle [3, 5]. To this end, several qualitative studies have suggested that participation in local community groups can lead to less stigmatisation of negatively defined others. Howarth [24], for instance, found that resistance to race-related stigma among black youth in England was developed through participation in community dialogues and relationships, made possible by membership in black supplementary schools. Looking specifically at HIV-related stigma, the Sonagachi Project, a community-based HIV intervention in the red light district of Kolkata, fostered increased community organization by female sex workers, leading to the women gaining more information about HIV, adding more value and acceptability to HIV prevention efforts [25], as well as enabling women to challenge the stigmatisation of commercial sex work [10]. Higher levels of social capital have been linked to greater likelihood of ‘moral behaviour’ by individuals [26, 41]. However, examination of the link between levels of social capital and stigma-related attitudes held by the general population towards those with a potentially stigmatizing condition (e.g., PLHIV) have not been investigated adequately.

We identified only two quantitative studies that explored the link between community group participation and HIV-related stigma. A study in a South African township [9] found that social capital, measured in terms of components including empowerment, trust and group membership, predicted levels of stigma above and beyond demographic covariates (e.g., age, gender, marital status) and whether the participant knew someone with HIV. Sivaram et al. [39] examined links between social capital and HIV stigma among commercial female sex workers and men who frequent beer halls in Chennai, India. They found that, among men and women, membership of formal community groups was associated with reduced fear of HIV transmission, reduced shame, blame and judgement, and reduced personal support for discriminatory actions against PLHIV. In addition, a sense of trustworthiness and the ability to rely on others for financial help were strongly associated with lower levels of stigma. Overall, the literature on links between social capital and stigma tends to be qualitative in nature with the few quantitative studies using cross-sectional survey designs.

In this paper, we use prospective data from a general population cohort survey in eastern Zimbabwe to describe patterns of association between community group participation and stigmatising attitudes and to test a possible explanatory framework.

## Explanatory Framework

A number of definitions have been advanced for stigma [17, 23, 30]. The presence of stigma within a society depends both on the extent to which individuals hold and express stigmatising attitudes and the extent to which individuals holding the devalued markers internalise these views [28]. Our focus here is on factors that can reduce stigmatising attitudes amongst community members; therefore, for the purposes of the current analysis, we define stigma as negative thoughts, feelings or actions towards people bearing some devalued marker (in this case having AIDS) [7].

Social capital has been defined as the community cohesion that results from positive aspects of community life [37] and is considered to have a ‘network’ dimension (high levels of participation in community groups) and a ‘norm’ dimension (particularly, levels of trust and reciprocity amongst community members). The former is generally considered to be a more powerful marker of social capital [12] and here we define social capital in terms of peoples’ participation in local community groups [8].

**Fig. 1:**
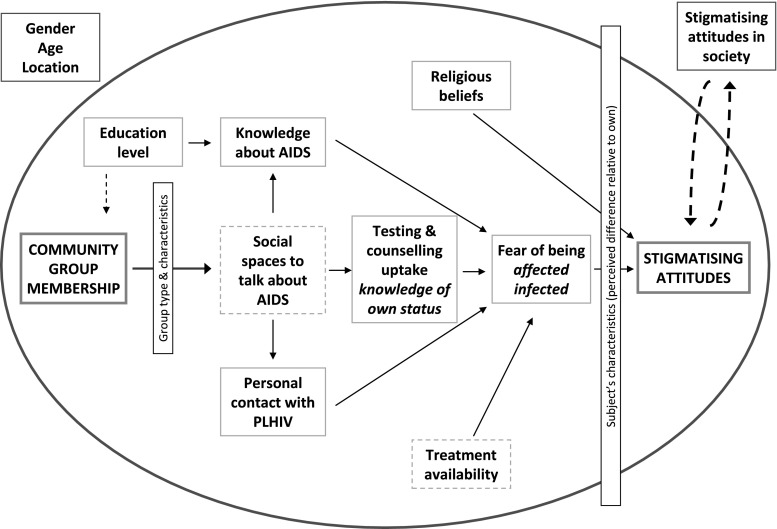
Explanatory framework for factors influencing the relationship between membership of community groups and reduced stigmatising attitudes towards people living with HIV and AIDS

An analytical framework for investigating the association between community group membership and stigmatising attitudes was developed from the literature [7, 15, 27] and is shown in Fig. 1. Underlying levels and patterns of stigma vary between societies according, for example, to differences in cultural and religious beliefs and availability of antiretroviral therapy. Within a given society, Parker and Aggleton [34] suggest that attitudes towards, for example, a specific HIV-infected individual will depend not only on perceived differences in HIV infection status (which may be altered following testing and counselling) but also on differences in varying combinations of devalued social markers such as gender, age, sexual orientation, class, race or ethnicity. Such relations of power and control create space for some groups to devalue others based on these differences. Equally, individuals in the same community may be more or less likely to hold stigmatising attitudes depending upon a number of factors including gender, age and education level.

Currently, there is a call for better understandings of “health enabling community contexts” [40], and more specifically “AIDS Competent Communities” [32], in which people are most likely to access HIV/AIDS services, and least likely to stigmatise the AIDS-afflicted. Various strands of the psychosocial research literature point to possible pathways between health-enabling communities and their potential health-enhancing effects. These community contexts are said to support the development of a health-enhancing reflection-action cycle, by providing opportunities for critical thinking about the obstacles to health and renegotiation of health-damaging attitudes and behavioural norms [13]. Ideally, such critical reflection and action also goes hand in hand with an increase in the development of health-related agency amongst previously disempowered groups [42]. Opportunities for critical thinking and empowerment are said to be fostered by the presence of ‘social spaces’ within which community members can discuss and debate HIV/AIDS-related attitudes and behavioural norms with liked and trusted peers [6]. Such discussion can, in turn, facilitate the development of less stigmatising attitudes [1, 33]. This could happen, for example, through the acquisition of improved knowledge about how HIV is transmitted and the symptoms of AIDS, through increased contacts with and familiarity with PLHIV and/or increased uptake of testing and counselling services leading to greater awareness of their HIV-infection status. The impact of a particular community group in reducing stigma will depend, in part, upon the nature of its primary activities and other characteristics including, critically, the extent to which it provides social spaces for people to engage in dialogue on HIV/AIDS and renegotiation of previously harmful behavioural and social norms and attitudes.

## Methods

### Data

The data used in the study were taken from the third and fourth rounds of a longitudinal population-based HIV/AIDS survey in Manicaland province, eastern Zimbabwe, where HIV prevalence fell from 23 % in 1998–2000 to 18 % in 2003–2005 [19]. The detailed procedures used in this survey have been published [18]. In brief, the data were collected in 12 locations (two small towns, two tea and coffee estates, two forestry plantations, two roadside trading settlements and four subsistence farming areas). A census of all households in each location was carried out in a phased manner (one site at a time) between July 2003 and August 2005 (round three) and again between July 2006 and November 2008 (round four). Men and women aged 15–54 years and resident within these households, at the date of the round three census (the baseline for the current study), were invited for an interview on a range of topics including socio-demographic characteristics, membership of community groups, knowledge about HIV/AIDS, personal knowledge of people with AIDS, uptake of HIV testing and counselling, and stigma, and tested for HIV infection. Ninety-six percent of households identified in the census were enumerated and 83 % of the men and women resident in these households agreed to participate in the individual survey. In round four (the follow-up survey for the current study), individuals resident in a random sample of two-thirds of households were selected as eligible for a further interview. 55 % of the individuals in these households who also had been interviewed at baseline (i.e., members of the closed cohort) were re-interviewed at follow-up. The principal reason for loss-to-follow-up was outmigration from the study areas with refusal rates in successive rounds have been in the range 1–2 % [19].

Ethical approval for the study was obtained from the Medical Research Council of Zimbabwe (Number MRCZ/A/681) and the St. Mary’s Local Research Ethics Committee in London, United Kingdom (HIV/GUM EC 03.66 R&D 03/SB/004E). Written informed consent was obtained from all participants. For participants aged below 18, this consent was obtained from the next of kin, carers or guardians on behalf of the minors or children in the study.

### Measures

Dichotomous variables were constructed for the independent (group membership) and dependent (stigmatising attitudes) variables. For the variables on overall group membership, respondents were treated as participating in community groups if they reported membership of at least one group that they regarded as functioning effectively [21]. For the variables on participation in specific types of groups (more details of these groups can be found in [21]), each respondent was treated as being a member of the group they said they spent the most time in. Survey respondents were treated as having stigmatising attitudes towards people living with HIV if they reported being unwilling to care for a relative with AIDS. We operationalised our definition of stigmatising attitudes in this way because this was the most suitable question on stigma contained in the Manicaland survey questionnaires. Practical constraints on individuals’ ability to care for a relative with AIDS also might have led respondents to report being unwilling to care for a relative with AIDS. We addressed this limitation in the data analysis by controlling for the practical constraints associated with living in the more cramped housing conditions found in urban and estate settings and the time constraints associated with being in formal sector employment.

In testing the proposed framework for explaining the association between community group membership and reductions in stigmatising attitudes, we constructed a variable indicating whether or not the group each respondent spent most time in provided social spaces for dialogue on HIV/AIDS. A group was considered to provide social spaces to talk about AIDS if: (1) meetings were held at least once a month; and (2) group members were said to advise each other on issues relating to caring for people living with HIV/AIDS in formal or informal discussions. Knowledge about HIV/AIDS was measured at baseline and at follow-up using indices constructed from responses to a series of questions about modes of transmission, protective measures and symptoms [22]. Respondents were treated as having good knowledge about HIV/AIDS at baseline if they scored 60 % or above on this index. A respondent’s knowledge was considered to have improved between baseline and follow-up if their index score had increased by 5 % or more. In assessing whether group membership was associated with greater personal contact with people living with HIV, respondents were treated as having personal contact with people living with HIV if they reported knowing at least one person with AIDS, other than a relative or work colleague, who lived in the same village or town. Finally, respondents were treated as having taken up voluntary counselling and testing services if they reported having had at least one HIV test between the two rounds of the survey.

### Data Analysis

The proportions of individuals participating in the survey at both rounds: (a) who were members of at least one community group throughout the study period; and (b) who joined a group during the inter-survey period were calculated. The socio-demographic characteristics of members of community groups then were compared with those of non-members using logistic regression. Tests for association between group membership and stigmatising attitudes were conducted using logistic regression; first, adjusting for sex and age only, and then also adjusting for other potential confounding factors—education, marital status, socio-economic status, formal sector employment, religion, location of residence, HIV infection status, history of caring for a person living with HIV (PLHIV), and stigmatising attitudes at baseline.

In investigating the proposed framework for explaining the association between community group participation and stigmatising attitudes (Fig. 1), odds ratios (adjusted for the potential confounding factors listed above) were calculated for stigmatising attitudes towards PLHIV amongst members of community groups compared to non-members for groups with different principal activities (types) and characteristics. The types of groups examined were church groups, women’s groups, cooperatives, farmers groups, burial societies, rotating credit societies (or savings clubs), youth groups, sports clubs, AIDS groups and political parties [21]. The characteristics of groups examined were whether the group has a single sex or mixed membership, whether the group interacts with other groups, whether alcohol is consumed during or after group meetings, whether the group receives external sponsorship, and whether group members discuss care for PLHIV formally or informally during their meetings. To assess whether improved knowledge about AIDS, greater personal contact with PLHIV, and increased uptake of counselling and testing services mediate the association between community group participation and stigmatising attitudes, logistic regression was used, first, to test for associations between group membership (throughout the study period) and these properties and, second, to test for associations between these properties and stigmatising attitudes. Finally, a logistic regression model was developed to establish whether the association between community group membership and stigmatising attitudes remained or weakened after adjusting for the proposed mediating factors.

All analyses were carried out in STATA version 10 (STATA Corp., College Station, TX, USA). In general, similar effects were found for males and females so the results are shown for both sexes combined. However, instances where effects differed by gender are noted in the text.

## Results

### Local Patterns of Community Group Membership

A third (36 %, 1,894/5,253) of the study participants reported membership of at least one community group at baseline and again at follow-up, and two-fifths (41 %, 996/2,398) of those not in a group at baseline joined a group between the two rounds of the survey. Compared to non-group members, individuals who participated in community groups were more likely to be female, to have secondary school education, to follow a Christian religion, and to have cared for a PLHIV, and less likely to be young (15–19 years of age), to be better off economically or to be in formal sector employment, and to be infected with HIV (Table 1). Those who joined a group between rounds of the survey generally had characteristics that were intermediate between those of long-term group members and non-members. The proportions expressing stigmatising attitudes towards PLHIV at baseline were 2.9 % (95 % CI 2.1–3.7 %), 5.8 % (4.5–7.5 %) and 8.7 % (7.3–10.3 %), respectively, for long-term group members, new joiners and non-members.

**Table 1:** Socio-demographic characteristics of study participants at baseline, by current and future membership of community groups, Manicaland, Zimbabwe

Characteristic at baseline	Group member at baseline and at follow-up			Joined a group between baseline and follow-up			Not a member at baseline or at follow-up	
	%	N	aOR^a^	%	N	aOR^a^	%	N
*Sex*								
Male	15.0	284	1	31.8	317	1	59.6	836
Female	85.0	1610	7.5***	68.2	679	3.1***	40.4	566
*Age*-*group*								
15–19 years	7.4	141	1	17.8	177	1	25.8	362
20–29 years	23.1	437	2.0***	34.9	348	1.4**	33.5	470
30–39 years	27.0	511	3.9***	24.0	239	1.6**	21.0	295
40–54 years	42.5	805	5.6***	23.3	232	1.5**	19.6	275
*Education level*								
Primary or less	49.0	928	1	37.7	375	1	38.9	546
Secondary or more	51.0	966	2.2***	62.3	621	1.8***	61.1	856
*Marital status*								
Never married	10.9	206	1	25.0	249	1	36.6	513
Married	71.1	1,346	1.1	60.8	606	1.0	52.0	729
Formerly married	18.1	342	0.7*	14.2	141	0.7	11.4	160
*Socio*-*economic status* ^*b*^								
Poorest tercile	36.7	696	1	39.3	391	1	33.8	474
Middle tercile	33.6	637	0.7**	33.2	331	0.7**	35.9	504
Least poor tercile	29.6	561	0.8**	27.5	274	0.7**	30.2	424
*Employment*								
No	87.4	1,656	1	78.5	782	1	73.7	1,033
Yes	12.6	238	0.6***	21.5	214	0.9	26.3	369
*Religion*								
None	1.8	35	1	10.4	104	1	16.8	235
Traditional	0.5	10	1.7	1.8	18	1.1	2.5	35
Christian	97.6	1,849	9.6***	87.8	874	1.5**	80.7	1,132
*Location of residence*								
Rural village	44.8	849	1	35.3	352	1	35.0	490
Roadside trading settlement	22.9	434	1.1	16.0	159	0.9	17.4	244
Estate	25.3	480	0.8*	31.9	318	1.0	30.9	433
Town	6.9	131	0.3***	16.8	167	0.9	16.8	235
*HIV status* ^*b*^								
Uninfected	84.5	1,600	1	81.6	813	1	85.1	1,193
Infected	15.5	294	0.7**	18.4	183	1.1	14.9	209
*Cared for PLHIV*								
No	59.3	1124	1	74.1	738	1	75.3	1,056
Yes	40.7	770	2.0***	25.9	258	1.1	24.7	346
*Stigmatising attitudes* ^*c*^								
No	97.1	1,840	1	94.2	938	1.0	91.3	1280
Yes	2.9	54	0.3***	5.8	58	0.7*	8.7	122

^a^Sex- and age-adjusted odds ratios (aOR) from logistic regression for difference versus non-members

^b^Measured at follow-up (2006–2008)

^c^Unwilling to care for a relative with AIDS at baseline (2003–2005)

* ~*p* < 0.05; ** ~*p* < 0.01; *** ~*p* < 0.001

### Association Between Participation in Community Groups and Stigmatising Attitudes at Follow-Up

Table 2 shows the results of the tests for association between community group membership and stigmatising attitudes (being unwilling to care for a relative with AIDS) at follow-up. Overall, 4.4 % of women and 8.5 % of men in the study expressed stigmatising attitudes towards PLHIV (test for difference by sex, age-adjusted odds ratio (aOR) = 0.53, *p* < 0.001). In the sex- and age-adjusted analysis, individuals aged over 20 years at baseline, those with secondary education, and those expressing willingness to care for a relative with AIDS at baseline each were significantly (*p* < 0.05) less likely to express stigmatising attitudes towards PLHIV at follow-up.

**Table 2:** Tests for association between membership of community groups and stigmatising attitudes (being unwilling to care for a relative with AIDS), Manicaland, Zimbabwe, 2003–2008

Characteristic at baseline (unless stated otherwise)	Unwilling to care for a relative with AIDS^a^		Sex- and age-adjusted	Fully adjusted^b^
	% (95% CI)	N	aOR	aOR
All respondents	5.8 (5.1–6.5)	4,292	–	–
*Community group membership*				
Not a member at baseline or at follow-up	9.5 (8.0–11.0)	1,402	1	1
Member at baseline and at follow-up	3.4 (2.6–4.2)	1,894	0.41***	0.46***
Joined group between baseline and follow-up	5.0 (3.7–6.4)	996	0.57**	0.62**
*Sex*				
Male	8.5 (7.0–9.9)	1,437	1	1
Female	4.4 (3.6–5.1)	2,855	0.53***	0.67*
*Age*-*group*				
15–19 years	9.1 (6.9–11.3)	680	1	1
20–29 years	5.2 (4.0–6.4)	1,255	0.59**	0.86
30–39 years	5.4 (4.0–6.7)	1,045	0.63*	1.09
40–54 years	4.9 (3.7–6.0)	1,312	0.62*	1.00
*Education level*				
Primary or less	5.9 (4.8–7.0)	1,849	1	1
Secondary or more	5.6 (4.7–6.6)	2,443	0.67**	0.81
*Marital status*				
Never married	9.1 (7.3–10.9)	968	1	1
Married	4.7 (3.9–5.5)	2,681	0.66	0.73
Formerly married	5.0 (3.3–6.7)	643	0.79	0.86
*Socio*-*economic status*				
Poorest tercile	5.5 (4.4–6.6)	1,561	1	1
Middle tercile	5.4 (4.2–6.5)	1,472	0.99	0.92
Least poor tercile	6.5 (5.1–7.9)	1,259	1.25	1.12
*Employment*				
No	5.8 (5.1–6.6)	3,471	1	1
Yes	5.4 (3.8–6.9)	821	0.77	0.85
*Religion*				
None	8.3 (5.5–11.1)	374	1	1
Traditional	12.7 (4.2–21.2)	63	1.73	1.72
Christian	5.4 (4.7–6.1)	3,855	0.73	0.87
*Location of residence*				
Rural village	5.6 (4.5–6.7)	1,691	1	1
Roadside trading settlement	6.7 (5.0–8.4)	837	1.19	1.19
Estate	4.9 (3.7–6.1)	1,231	0.81	0.80
Town	6.8 (4.6–8.9)	533	1.18	1.10
*HIV status (at follow*-*up)*				
Uninfected	6.0 (5.2–6.8)	3,476	1	1
Infected	4.7 (3.2–6.1)	816	0.87	0.88
*Cared for PLHIV (prior to baseline)*				
No	6.0 (5.2–6.9)	2,918	1	1
Yes	5.2 (4.0–6.8)	1374	0.91	1.04
*Unwilling to care for relative with AIDS*				
No	5.1 (4.4–5.8)	4,058	1	1
Yes	17.5 (12.6–22.4)	234	3.62***	3.15***

^a^Measured at follow-up; comparison group—individuals who were not members of community groups either at baseline or at follow-up

^b^aOR, odds ratio calculated using logistic regression adjusting for the effects of all other characteristics

* ~*p* < 0.05; ** ~*p* < 0.01; *** ~*p* < 0.001

Stigmatising attitudes towards PLHIV at follow-up were significantly less common amongst long-term members (age-adjusted OR = 0.41, 95 % CI, 0.29–0.57) and new members (0.57, 0.40–0.80) of community groups than amongst non-members (Table 2). These associations reduced only slightly and remained statistically significant in the fully-adjusted model.

### Effects of Different Types and Characteristics of Community Groups on Stigmatising Attitudes

**Fig. 2:**
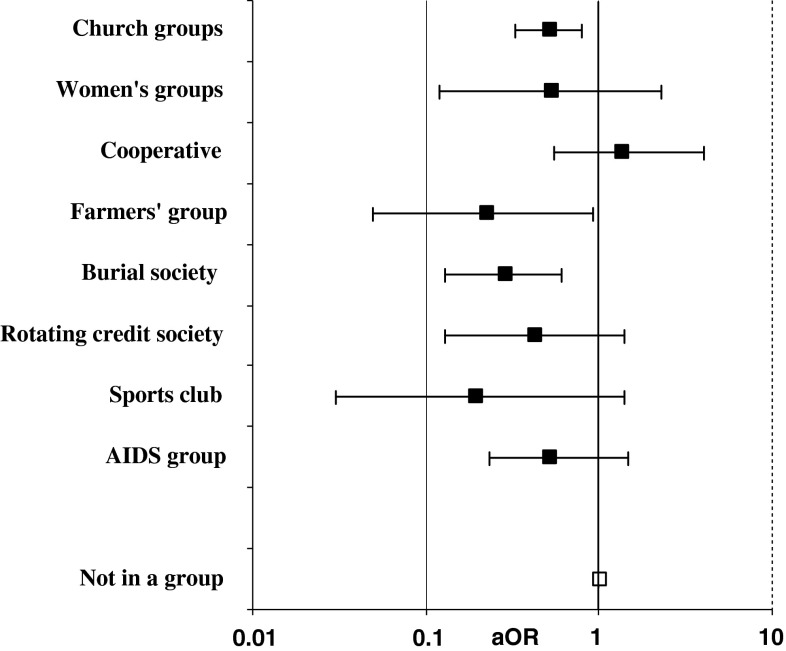
Associations between community group membership, at baseline and at follow-up, and stigmatising attitudes towards people living with HIV infection (being unwilling to care for a relative with AIDS) at follow-up, by type of group

Church groups were the type of community group that the greatest numbers of individuals reported spending most time in (24 %). These were followed by burial societies (8 %), farmers groups (2.5 %), AIDS groups (2 %) and rotating credit societies (2 %). Figure 2 shows the patterns of association between community group membership (at both baseline and follow-up) and stigmatising attitudes at follow-up based on the types of groups that men and women in the study reported spending most time in. All forms of community group, except for cooperatives, showed protective associations, with the associations for church groups, farmers groups and burial societies being statistically significant.

For men and women combined, community group membership is associated with lower levels of stigmatising attitudes irrespective of any of the group characteristics investigated (Table 3). The same patterns were found for women alone. However, for men, protective associations were observed in mixed sex groups (aOR = 0.39, *p* = 0.011) but not in single sex groups (aOR = 0.80, *p* = 0.7), in groups that interacted with other groups (aOR = 0.45, *p* = 0.044) but not in those that didn’t meet with other groups (aOR = 0.48, *p* = 0.14), in groups that consumed alcohol (aOR = 0.34, *p* = 0.025) and but in those that didn’t (aOR = 0.60, *p* = 0.2), and in groups that did not discuss care for PLHIV (aOR = 0.37, *p* = 0.042) but not in those that did discuss care for PLHIV (aOR = 0.55, *p* = 0.13).

**Table 3:** Types and characteristics of community groups associated with stigmatising attitudes towards PLHIV at follow-up, Manicaland, Zimbabwe

Characteristic of group at baseline	Member of group with characteristic			Member of group without characteristic		
	%	N	aOR (95 % CI)^a^	%	N	aOR^a^
Single sex membership	3.1	957	0.40 (0.25–0.65)	3.6	937	0.42 (0.27–0.65)
Interacts with other groups	3.1	1398	0.38 (0.25–0.58)	4.0	496	0.50 (0.29–0.85)
Alcohol consumed at meetings	2.8	633	0.33 (0.19–0.57)	3.6	1,261	0.46 (0.30–0.70)
Sponsored	2.8	669	0.36 (0.21–0.61)	3.7	1,225	0.44 (0.29–0.67)
Discuss care for PLHIV	3.6	1107	0.44 (0.29–0.69)	3.0	787	0.37 (0.23–0.61)
Reference—not a group member	–	–	–	9.5	1,402	1

^a^aOR: odds ratio for stigmatising attitudes at follow-up (2006–2008), for members of community groups (at baseline and at follow-up) with and without characteristic (at baseline) versus non-group members, adjusted for sex, age-group, education, marital status, socio-economic status, employment, religion, location of residence, HIV infection, history of caring for PLHIV, and stigmatising attitude at baseline

### Factors Hypothesised to Mediate the Association Between Participation in Community Groups and Stigmatising Attitudes

Overall, community group membership (at both rounds) was not associated with better knowledge about AIDS at baseline or with greater improvement in knowledge over the inter-survey period (Table 4). However, group membership showed a weak negative association with knowledge about AIDS at baseline for men (aOR = 0.77, *p* = 0.08) and a weak positive association for women (aOR = 1.23, *p* = 0.08). Community group members were more likely than non-members to report knowing a non-relative with AIDS at baseline and, amongst those who did not, to report knowing a non-relative with AIDS at follow-up (*p* = 0.08). Community group members also were more likely than non-members to report having taken up HIV testing and counselling services during the inter-survey period.

**Table 4:** Associations between community group membership (at baseline and follow-up) and potential mediating factors and stigmatising attitudes (at follow-up), Manicaland, Zimbabwe, 2003–2008

Mediating factor	Knowledge about AIDS		Knowledge of non-relative with AIDS		HIV testing and counselling between baseline and follow-up	Unwilling to care for a relative with AIDS (at follow-up)	
	At baseline	Improved between baseline and follow-up	At baseline	At follow-up but not at baseline		Model adjusted for single factor	Model adjusted for all factors
	aOR	aOR	aOR	aOR	aOR	aOR	aOR
*Community group membership*							
At baseline and at follow-up	1.02	1.05	1.51***	1.22	1.54***	0.46***	0.43***
*Knowledge about AIDS*							
At baseline	–	–	–	–	–	0.71*	0.77
Improved between baseline and follow-up	–	–	–	–	–	0.79	0.82
*Knowledge of non*-*relative with AIDS*							
At baseline	–	–	–	–	–	0.87	0.95
At follow-up but not at baseline	–	–	–	–	–	0.62*	0.66
HIV testing and counselling							
Between baseline and follow-up	–	–	–	–	–	0.69	0.77

aOR: odds ratio for stigmatising attitudes for members of community groups (at baseline and at follow-up) versus non-group members, adjusted for age-group, education, marital status, socio-economic status, employment, religion, location of residence, HIV infection, history of caring for PLHIV, and stigmatising attitude at baseline

N = 3,296

* ~*p* < 0.05; ** ~*p* < 0.01; *** ~*p* < 0.001

Knowledge about AIDS, recent knowledge of a non-relative with AIDS, and recent uptake of HIV testing and counselling services (*p* = 0.08) were each associated with less stigmatising attitudes towards PLHIV (Table 4). HIV testing and counselling was associated with less stigmatising attitudes towards PLHIV for men (aOR = 0.30, *p* = 0.048) but not for women (*p* = 0.6). However, adjustment for these factors did not reduce the association between community group membership and less stigmatising attitudes.

## Discussion

In predominantly rural areas in Manicaland province in eastern Zimbabwe where, as elsewhere in the country, HIV prevalence has been declining but remains at a high level [18, 20], and at time when availability of ART services was still low, we found that men and women who participated in community groups were less likely than their peers to have stigmatising attitudes towards PLHIV. This was the case at baseline and also 3 years later at follow-up when the association remained statistically significant even after adjusting for pre-existing levels of stigma. As might be expected, members of AIDS clubs and church groups were significantly less likely to have stigmatising attitudes towards PLHIV than men and women who were not members of community groups and similar trends were seen in a wide range of different types of groups.

In the study, we also developed and tested a new explanatory framework derived from the literature. However, the results of the prospective analysis reported here provided little evidence to support the pathways between community group participation and reduced stigmatising attitudes hypothesised in the framework. Overall, the effects of group membership on stigma did not appear to differ greatly by type of group activity or by other group characteristics. However, for men, the picture was more complex with mixed sex groups and groups that interacted with other groups being more beneficial but alcohol consumption and absence of discussion about AIDS also associated with lower levels of stigma. Whilst knowledge about HIV/AIDS, uptake of HIV testing services, and exposure to PLHIV were found to be associated with less stigmatising attitudes, controlling for these attributes did not reduce the association between community group membership and stigmatising attitudes towards PLHIV, which suggests that they may not be important mechanisms through which group membership facilitates reductions in stigma.

There may be other characteristics (including those that are difficult to measure in surveys) that affect whether community groups provide positive or negative social capital in combatting stigma. In addition, some of the characteristics that we did examine may have mixed effects. For example, in qualitative work conducted in the same study areas in Zimbabwe, we found that discussions about AIDS within groups sometimes had detrimental effects in spreading incorrect information [38]. Nevertheless, the finding that stigmatising attitudes towards PLHIV were not lower amongst individuals who participated in community groups that provided social spaces for discussion about care for PLHIV than amongst those in groups that did not discuss AIDS was unexpected. In an earlier analysis of the cross-sectional data collected at baseline, we found that members of groups that provided these spaces were especially unlikely to have stigmatising attitudes towards PLHIV—whilst men and women who participated in groups that did not provide social spaces for discussions about HIV and AIDS had similar levels of stigmatising attitudes to those who did not participate in community groups at all, those who were members of groups that did discuss AIDS were less likely to express stigmatising attitudes (men: 3.25 vs. 6.3 %, aOR = 0.56, *p* = 0.002; women: 3.7 vs. 8.1 %, aOR = 0.57, *p* < 0.001) [31].

These contrasting findings regarding the beneficial effects of membership of groups that provide social spaces for discussion about AIDS may be because the effects of these social spaces change over time as HIV epidemics progress. The severe economic and political instability that occurred in Zimbabwe between the two survey rounds also may have disrupted the nature and quality of group discussions about AIDS and could have affected attitudes towards PLHIV more generally. The contrast in findings also may have resulted from the self-selective nature of participation in community groups, particularly where people with more progressive attitudes are most likely to join these groups. In the current data from Zimbabwe, individuals who joined community groups during the inter-survey period had lower levels of stigmatising attitudes at baseline (i.e., prior to joining a community group) than those who did not join a group. This suggests that the associations observed between community group membership and lower stigma may be partly due to selection. However, it should be noted that these associations remained substantial and statistically significant after adjusting for multiple potential confounding factors including education, employment, religion and pre-existing attitudes towards PLHIV. Furthermore, the individuals who joined community groups during the inter-survey period had higher levels of stigma at baseline than those who were already participating in community groups (*p* = 0.01).

This study was based on prospective survey data from a large general population sample and included detailed data on a range of different types of community participation. However, the study also has a number of limitations. The variable used to represent stigmatising attitudes was based on the most relevant question asked in the Manicaland study and reflects a single aspect of a complex multi-dimensional construct [30]. Furthermore, study participants who reported being unwilling to care for a relative with AIDS may have expressed this view due to practical obstacles. Those who lived in estates and, to a lesser extent, in towns were more likely to report being unwilling to care for a relative with AIDS possibly due to constrained living conditions. However, this was not the case for participants in formal sector employment (who may have less time to care for sick relatives) and the statistical association between community group membership and reduced stigma remained after differences in residence were controlled for in the analysis. Relatively few men and women reported stigmatising attitudes towards PLHIV. This may have been because of the narrow operational definition used in the study but also could reflect social desirability bias which might possibly have been greater amongst community group members. Attrition in the cohort over the 3 years inter-survey period was quite high, although the great majority of those not seen again at follow-up no longer lived in the study communities.

Social capital has been found to be helpful in HIV prevention [8, 21, 36] but we found only two previous studies on its effects on stigma. In South Africa and India, respectively, Chiu et al. [9] and Sivaram et al. [39], also found social components to be associated with less stigmatising attitudes towards PLHIV. As far as we are aware, the current study is the first to provide evidence on the pathways that may link social capital and stigma. This is important because an understanding of how community groups can reduce stigma could be helpful in identifying strategies for strengthening and expanding their role.

This study provides further evidence for an association between community group membership and reduced stigma. However, the mechanisms remain unclear and the association may result mainly from selective community participation amongst more progressive individuals rather than from a beneficial effect of group membership. More research is needed to clarify the nature of the association as well as to assess the impact of increasing ART availability. Nevertheless, large numbers of male and female residents in eastern Zimbabwe report participating in community group activities and these groups may have contributed to reducing overall levels of stigma. Efforts to provide support for community groups (Pronyk et al. [35]), to promote and inform discussions about HIV/AIDS within group meetings, and to engage local community groups as partners in anti-stigma programmes could be effective means of reducing current levels of stigma.

## References

[1] Baum F (1998). The new public health: An Australian perspective.

[2] Bond V, Nyblade L (2006). The importance of addressing the unfolding TB-HIV stigma in high prevalence settings. Community and Applied Social Psychology.

[3] Breakwell GM (1986). Coping with threatened identities.

[4] Brewer M, Gardner W (1996). Who is this “We”? Levels of collective identity and self representations. Journal of Personality and Social Psychology.

[5] Campbell C, Deacon H (2006). Unravelling the contexts of stigma: From internalisation to resistance to change. Journal of Community and Applied Social Psychology.

[6] Campbell C, Gibbs A, Nair Y, Maimane S (2009). Potential false promise or complicated possibilities? Empowerment and participation amongst female health volunteers in South Africa. Health Management.

[7] Campbell, C., Nair, Y., Maimane, S., & Nicholson, J. (2007). “Dying twice”: A multi-level model of the roots of AIDS stigma in two South African communities. *Journal of Health Psychology*, *12*(3), 403–416. 10.1177/1359105307076229 17439992

[8] Campbell C, Williams B, Gilgen D (2002). Is social capital a useful conceptual tool for exploring community level influences in HIV infection? An exploratory case study from South Africa. AIDS Care.

[9] Chiu J, Grobbelaar J (2008). HIV-related stigma and social capital in South Africa. AIDS Education and Prevention.

[10] Cornish F (2006). Challenging the stigma of sex work in India: Material context and symbolic change. Journal of Community and Applied Social Psychology.

[11] Crosby RA, Holtgrave DR (2003). Social capital as a predictor of adolescents’ sexual risk behavior. AIDS and Behaviour.

[12] Foley M, Edwards B (1999). Is it time to divest in social capital?. Journal of Public Policy.

[13] Freire P (1973). Education for critical consciousness.

[14] Genberg BL, Kawichai S, Chingono A, Kilonzo GP (2008). Assessing HIV/AIDS stigma and discrimination in developing countries. AIDS and Behaviour.

[15] Gillies P (1998). The effectiveness of alliances and partnerships for health promotion. Health Promotion International.

[16] Gittens, B. (2008). *AIDS in Zimbabwe: Disrupting the community’s social fabric*. United Methodist Committee on Relief, Health and Welfare Ministries.

[17] Goffman E (1963). Stigma: Notes on the management of spoiled identity.

[18] Gregson S (2006). HIV decline associated with behaviour change in eastern Zimbabwe. Science.

[19] Gregson S (2007). A critique of early models of the demographic impact of HIV/AIDS in sub-Saharan Africa based on empirical data from Zimbabwe. Proceedings of the National Academy of Sciences.

[20] Gregson S (2010). HIV decline due to reductions in risky sex in Zimbabwe? Evidence from a comprehensive epidemiological review. International Journal of Epidemiology.

[21] Gregson S (2011). Social capital and reduced female vulnerability to HIV infection in rural Zimbabwe. Population and Development Review.

[22] Gregson S, Zhuwau T, Anderson R, Chandiwana S (1998). Is there evidence for behaviour change in response to AIDS in rural Zimbabwe?. Social Science and Medicine.

[23] Heatherton T, Kleck R, Hebb M, Hull J (2003). The social psychology of stigma.

[24] Howarth C (2006). Race as stigma: Positioning the stigmatized as agents, not objects. Journal of Community and Applied Social Psychology.

[25] Jana S, Basu I, Rotheram-Borus M-J, Newman P (2004). The Sonagachi Project: A sustainable community intervention program. AIDS Education and Prevention.

[26] King PE, Furrow JL (2004). Religion as a resource for positive youth development: Religion, social capital, and moral outcomes. Development Psychology.

[27] Kreuter, M., Lezin, N., & Koplan, B. A. (1997). *National level assessment of community health promotion using indicators of social capital* (pp. 1–21). WHO/EURO Working Group on Evaluating Health Promotion Approaches.

[28] Link BG, Phelan JC (2001). Conceptualising stigma. Annual Reviews of Sociology.

[29] Low-Beer D, Stoneburner RL (2004). AIDS communication through social networks: Catalyst for behaviour change in Uganda. African Journal of AIDS Research.

[30] Mahajan AP (2008). Stigma in the HIV/AIDS epidemic: A review of the literature and recommendations for the way forward. AIDS.

[31] Nhamo M (2011). The role of churches in tackling HIV stigma in eastern.

[32] Nhamo M, Campbell C, Gregson S (2010). Contextual determinants of HIV prevention programme outcomes: Obstacles to local-level AIDS competence in rural Zimbabwe. AIDS Care.

[33] Ogden, J., & Nyblade, L. (2005) *Common at it’s core: HIV*-*related stigma*. International Research on Women (ICWR).

[34] Parker R, Aggleton P (2003). HIV and AIDS-related stigma and discrimination: A conceptual framework and implications for action. Social Science and Medicine.

[35] Pronyk PM, Harpman T (2008). Can social capital be intentionally generated? A randomised trial from South Africa. Social Science and Medicine.

[36] Pronyk PM, Harpman T (2008). Is social capital associated with HIV risk in rural South Africa?. Social Science and Medicine.

[37] Putnam RD (2000). Bowling alone: The collapse and revival of american community.

[38] Scott K, Campbell C, Gregson S, Nhamo M, Nyamukapa CA (2011). In what way do formal community groups impact HIV-related behaviours? The role of social capital in building HIV competence in rural Zimbabwe Social capital and AIDS competent communities: Evidence from eastern Zimbabwe vol Technical Report 3.

[39] Sivaram S, Zelaya C, Srikrishnan AK, Latkin C, Go VF, Sol S (2009). Associations between social capital and HIV stigma in Chennai, India. AIDS Education and Prevention.

[40] Tawil O, Verster A, O’Reilly K (1995). Enabling approaches for HIV/AIDS promotion: Can we modify the environment and minimize risk?. AIDS.

[41] Uslaner EM (1999). Trust but verify: Social capital and moral behaviour. Social Science Information.

[42] Wallerstein N (1992). Powerlessness, empowerment and health: Implications for health promotion programmes. American Journal of Health Promotion.

